# An Optimization Approach for the Production of High-Purity Vitamin C‑Nicotinamide Cocrystals by the Gas Antisolvent (GAS) Technique with CO_2_ and Ethanol

**DOI:** 10.1021/acsomega.5c08253

**Published:** 2026-01-21

**Authors:** Clóvis A. Balbinot Filho, Thayli R. Araujo, Jônatas L. Dias, Evertan A. Rebelatto, Adailton J. Bortoluzzi, Mariana M. Vernaschi, Tânia B. Creczynski-Pasa, Sandra R. S. Ferreira, Marcelo Lanza

**Affiliations:** † Department of Chemical and Food Engineering, 28117 Federal University of Santa Catarina, Florianopolis 88040-900, Brazil; ‡ Department of Chemistry, 28117 Federal University of Santa Catarina, Florianopolis 88040-900, Brazil; § Department of Pharmaceutical Sciences, Federal University of Santa Catarina UFSC, Florianópolis, SC 88040-900, Brazil

## Abstract

Vitamin C (l-ascorbic acid, ASC) is a powerful antioxidant nutrient with diverse metabolic functions, regenerative properties, and anticancer potential. However, it is a highly unstable molecule. ASC can form a cocrystal with the amide of vitamin B_3_ (nicotinamide, NIC) through self-complementary hydrogen bonding, therefore improving its physical stability. Pressurized carbon dioxide (CO_2_), via the gas antisolvent (GAS) method, makes an excellent medium for cocrystallizing vitamins, particularly from ethanolic solutions. However, the controllable variables of the GAS method should be optimized for a feasible process. The production of the ASC:NIC cocrystal was optimized using a Box–Behnken experimental design (BBD) at 90 bar and with ethanol as the solvent while varying the temperature, CO_2_ flow rate, and ASC:NIC molar ratio. The final ASC and NIC contents in the cocrystals were determined by derivative spectrophotometry and supported by HPLC and elemental analysis. PXRD and DSC confirmed that high-purity (>99%) cocrystals can be produced by setting a proper initial molar ratio of starting compounds. The maximum cocrystal yield by GAS (85.2%) was attained at the optimized condition using a lower pressure (80 bar) due to higher supersaturation of the system. Purest cocrystals exhibited a needle-like morphology, fine particle size, and thermal stability while preserving the antioxidant power of ASC with high crystallinity and displaying no cytotoxicity to healthy epithelial cells up to 0.5 mM. GAS with CO_2_/ethanol could be optimized to overcome the solubility discrepancies between ASC and NIC in ethanol, producing vitamin C cocrystals at higher yields with a marked potential for nutritional and pharmaceutical applications.

## Graphical abstract

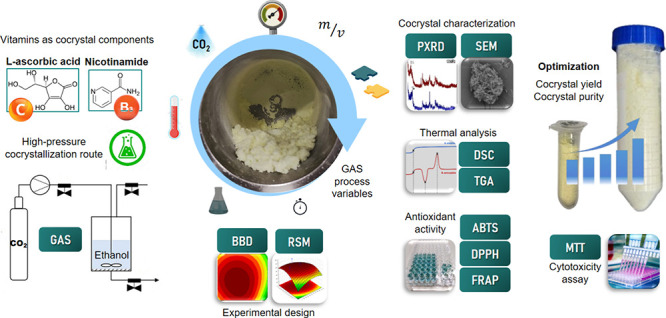

## Highlights

- ASC and NIC form a noncongruently soluble pair for cocrystallization in CO_2_/ethanol.
- The molar ratio of compounds fed in solution to GAS greatly affects cocrystal purity.
- Box–Behnken design with RSM and desirability profiler defined the best GAS conditions.
- Yield was increased with optimized conditions at 80 bar and a 1:10 mass-to-volume ratio.
- Cocrystals presented *in vitro* antioxidant activity and no cytotoxicity to endothelial cells.

## 1 Introduction

Vitamins are a group of biologically active organic compounds that are essential for health and are generally not synthesized by the human body.^1^ Vitamin C (l-ascorbic acid, ASC) is an essential nutrient that prevents scurvy and serves as a powerful antioxidant, playing a crucial role in maintaining a healthy organism through diverse biological functions, including cancer prevention,^2^ beyond its function as an antioxidant additive and antibrowning agent for foods.^3^ On the other hand, nicotinamide (NIC) is the amide form of niacin (vitamin B_3_), a dietary metabolite used to treat pellagra disease that is involved in the production of nicotinamide adenine dinucleotide (NAD+).^1,4^ The two vitamins interact through charge-transfer hydrogen bonds, forming a yellow nonsalt complex at a 1:1 stoichiometry.^5–7^ This complex was later regarded as a cocrystal.^8,9^

The ASC-NIC cocrystal exhibited outstanding features, including decreased viscosity and improved flowability compared to other vitamin combinations. The enhanced chemical stability of vitamin C, which is prone to oxidation, was achieved after cocrystallization with NIC.^10^ Consequently, the antiscorbutic property of vitamin C was maintained.^11^ It also overcame the poor compressibility of vitamin C, showing excellent tableting properties.^9^ Therefore, the ASC-NIC cocrystal is a relatively simple yet promising vitamin combination, potentially offering greater effectiveness in diverse applications compared to the vitamins alone.

ASC-based cocrystals have been obtained so far through mechanochemistry techniques such as neat or liquid-assisted grinding, ball milling, resonant acoustic mixing or twin-screw extrusion, and by gel-assisted or solution cocrystallization (solvent evaporation, cooling, and slurrying) methods.^8,9,12–15^ Recently, pressurized carbon dioxide (CO_2_) was successfully employed for the first time in the cocrystallization of ASC and NIC, by means of the gas antisolvent (GAS) method.^16,17^ However, there is limited discussion regarding product purity, presence of impurities, and ultimately, their suitability for food-like applications. Moreover, the susceptibility of ASC to oxidation in solution and its high solubility in polar solvents make traditional solution-based methods for cocrystal preparation unsuitable.^15^

Alternative cocrystallization techniques that achieve a more sustainable footprint, in line with green chemistry concepts, are preferred over traditional crystallization methods that employ toxic solvents, high temperatures, and prolonged periods for producing food-related cocrystals. Despite the safety concern, using compressed (sub- or supercritical) CO_2_ offers several advantages in producing high-purity cocrystals of thermolabile compounds at lower temperatures with no residual solvent, enabling control of the polymorphism^17^ and particle size distribution.^18^

Cocrystals produced by the semicontinuous GAS method are made of compounds that are sparingly soluble in CO_2_ (the antisolvent). During pressurization, CO_2_ promotes the volumetric expansion of the liquid phase. As the CO_2_ diffuses, the solubility of both coformers decreases sharply, inducing a controlled supersaturation and concomitant nucleation and coprecipitation of the cocrystal.^19,20^ Moreover, the inherent properties of CO_2_ (low toxicity, stability, availability, and ease of reuse) make the GAS method with ethanol as the solvent a sustainable route for the cocrystallization of water-soluble vitamins such as ASC and NIC.

Ethanol is the greenest organic solvent after water to produce food-grade cocrystals through solution-based recrystallization. On the other hand, the coformer NIC exhibits high solubility in organic solvents, such as ethanol and methanol, which tends to cause solubility improvements.^21^ Additionally, its solubility in the CO_2_/ethanol mixture at high pressures is also non-negligible, posing another obstacle to feasible cocrystallization by GAS. ASC-NIC cocrystals were previously synthesized by GAS with ethanol at fixed temperature and pressure, at varying starting ASC to NIC molar ratios.^17^ However, the maximum mass yield of precipitates was about 60%, and the impact of other variables is unknown. In this sense, addressing the common problem of solubility noncongruence between components in solution recrystallization^22–26^ is also a topic of attention.

Nonetheless, cocrystal synthesis by GAS involves a series of process variables that need to be controlled, such as pressure, temperature, antisolvent flow rate, coformer ratio, solvent, solution concentration, and volume,^27^ in which their direct and interaction effects are pivotal to elucidate how to control the cocrystallization process.^28–30^ The Design of Experiment (DoE) implies a systematic identification of parameter interaction and is highly recommended for process optimization.^27^ Optimizing GAS can overcome issues related to the thermodynamic solubility discrepancy of the cocrystal compounds in the selected solvent, and low precipitation yields,^31,32^ thereby achieving a cocrystal with desirable purity and yield.

Researchers have yet to fully explore the use of compressed CO_2_ in the synthesis of ASC cocrystals. Although the FDA has approved ASC-NIC cocrystals for human consumption,^33^ the potential for their use in novel food applications remains largely unknown. Therefore, this paper intends to (i) produce 1:1 ASC-NIC cocrystals by an alternative sustainable route: GAS method with CO_2_, applied to solutions with ethanol as a green solvent for solubilizing the coformers (ASC and NIC); (ii) employ optimization tools to convey the highest purity of the formed cocrystals, with minimal material loss; and (iii) investigate the aspects related to thermal stability and biological *in vitro* potential under optimized conditions to assess the impact of synthesizing ASC-NIC cocrystals by GAS on the vitamin’s functionality.

## 2 Materials and Methods

### 2.1 Chemicals

Cocrystal components l-ascorbic acid (ASC, 0.990 Sigma-Aldrich) and coformer nicotinamide (NIC, 0.990 Sigma-Aldrich) were used as received. Carbon dioxide (CO_2_, 0.999 molar fraction purity, White-Martins S.A.) was used as the antisolvent, and ethanol (0.998 molar fraction purity, Neon Comercial) served as the solvent for ASC and NIC. The standard antioxidant Trolox (6-hydroxy-2,5,7,8-tetramethylchroman-2-carboxylic acid), as well as the radicals (ABTS and DPPH) and the reagents used in antioxidant activity assays, were all of analytical grade. All reagents used in high-performance liquid chromatography were HPLC-grade.

### 2.2 Solubility Determination

Solubility measurements of the cocrystal components in pure ethanol were performed using the saturation shake-flask method.^34,35^ Excess amounts of ASC and NIC were individually added to ethanol in 50 mL sealed Erlenmeyer flasks and incubated at 25 °C, 35 °C, 45 °C, and 55 °C (±0.1 °C) under agitation at 100 rpm (Dubnoff, 304-D Ethink, Brazil) for 24 h. The equilibration time (no agitation) was 2 h.^36^ After solid decantation, a 1 mL aliquot of the supernatant was withdrawn with warmed pipettes. Then, the samples were filtered through a 0.45 μm hydrophilic PTFE syringe filter and diluted in ethanol as needed, ensuring they fit within the established calibration curves for ASC and NIC. The concentrations of ASC and NIC were determined in triplicate using a UV-spectrophotometer (PG Instruments, UK) at 246 nm (ASC) and 262 nm (NIC). Solubility was expressed as molar fractions, along with the standard deviation.

### 2.3 Cocrystallization by Gas Antisolvent (GAS)

The GAS experiments were conducted in a self-assembled high-pressure unit described in detail elsewhere.^37,38^ In short, it comprises a 0.6 L jacketed stainless steel cell connected to a series of syringe pumps (Teledyne ISCO Pump Model 500D, Lincoln, USA), a CO_2_ reservoir, and essential instrumentation, including valves, pressure transducers, and thermocouples.

Before processing , ASC and NIC are weighed at the desired molar ratio and dissolved in ethanol at the desired concentration using an ultrasonic bath until complete dissolution, and then filtered through a 0.45 μm hydrophilic polytetrafluoroethylene (PTFE) syringe filter. For each GAS assay, a different ASC/NIC solution was injected into the vessel to evaluate the effect of different GAS parameters on the properties of the produced cocrystals. Cocrystallization was carried out by the GAS method in four sequential steps: (*1) pressure equilibration; (2) system pressurization; (3) system stirring, and (4) product drying.*

*Pressure Equilibration:* To prevent pipe clogging due to the Joule-Thomson effect, CO_2_ was slowly introduced into the assembled vessel. The outlet valve remained closed until the internal pressure matched that of the CO_2_ reservoir, typically around 6 MPa. *System Pressurization:* following equilibration, compressed CO_2_ (20 MPa) was continuously pumped into the vessel at a constant flow rateundermagnetic stirring until the desired working pressure and temperature were achieved. *System Stirring:* The CO_2_ inlet valve was closed and the system then continued stirring for 10 min, maintaining constant pressure and temperature. This step was crucial for thorough phase mixing, which triggered the precipitation of cocrystals. *Product Drying:* Finally, with the outlet valve open, an additional 800 mL of CO_2_ was continuously pumped into the vessel. The process was done under the same temperature, pressure, and flow rate conditions as before, effectively removing any solvent that might have been adsorbed by the samples or present in the headspace.

Afterward, the system is depressurized, the chamber is opened, and solvent-free bulky powder is collected for further characterization and confirmation of cocrystal formation.

### 2.4 Design of Experiment (DoE)

A three-level factorial Box–Behnken design (BBD) with surface response methodology (RSM) was employed to investigate the effect of GAS parameters temperature (*T*, 35–55 °C), CO_2_ flow rate (*F,* 0.3–0.9 L·h^–1^) and ASC:NIC molar ratio (*M*, 1:2, 1:1 and 2:1) on the cocrystals’ purity, yield, and mean length. BBD experiments were held at 90 bar pressure, and conditions were chosen based on previous works on the cocrystallization of bioactives through GAS.^37–39^

#### 2.4.1 Response Variables

The three dependent variables (responses) for BBD (i) cocrystal purity (eq 1), (ii) cocrystal yield (eq 2), and (iii) cocrystal mean length were selected to assess the viability of the process and the size reduction effect on the particles. Cocrystal purity and yield were calculated based on the cocrystal mass obtained from GAS, where m_collected_ is the total mass of recovered precipitate from a unique GAS batch, and m_ASC,_ m_NIC_ refer to each compound’s initial masses. The particles’ length was determined by analyzing SEM micrographs (see item 2.5.3).

$$Cocrystal\;purity\;(\%)=\frac{m_{cocrystal(1:1)}}{m_{collected}}\times 100$$ 1

$$Cocrystal\;yield\;(\%)=\frac{m_{cocrystal(1:1)}}{(m_{ASC}+m_{NIC}{)}_{1:1}}\times 100$$ 2

The resulting cocrystal mass (m_cocrystal (1:1)_) used in eqs 1 and 2 were obtained following the mass balance applied for the GAS process according to works by Neurohr et al.,^40,41^ adapted to the ASC-NIC cocrystal,^16^ according to eq 3, where m_ASC, total_ and m_NIC, total_ are the final contents of ASC and NIC in the resulting samples, determined by the first-order derivative spectrophotometric (FODS) method recently validated for this cocrystal^16^ or HPLC, *R* is the cocrystal molar ratio (1.0) and *M* the molecular masses.

$$m_{cocrystal\;(1:1)}=m_{collected}-m_{ASC,total}+m_{NIC,total}\times R\times \frac{M_{ASC}}{M_{NIC}}$$ 3

Two assumptions were made regarding the ASC-NIC cocrystallization result by GAS to get to eq 3: (i) the cocrystal produced is at the 1:1 stoichiometry, as previously validated^17^ and (ii) only ASC can precipitate as an excess homocrystal in the bulk powder (i.e., all NIC present in the samples is from the cocrystal). The reasoning behind these assumptions is discussed later, and the equations and simplifications leading to eq 3 can be found elsewhere.^16,40^ Nonetheless, crystallographic (PXRD) and thermal (DSC) data (Supporting Information, Figures S1 and S2) support these findings.

#### 2.4.2 Optimization

The independent variables and responses were correlated using RSM, which generated quadratic response surfaces and a model for each response.^42^ A desirability profiler was built using Statistica v.12 software (StatSoft, Tulsa, USA), and the factors (temperature, CO_2_ flow rate, and molar ratio) were set to simultaneously maximize the cocrystal’s purity and yield while minimizing particle length, providing the condition to achieve the desired response. GAS runs performed at the optimum operational point (OP) validated the generated models by comparing experimental responses with predicted ones.

Further, additional GAS runs were performed at OP at a lower pressure (80 bar) and with increasing scales of starting compounds (1 to 10 mmol) in ethanol and higher solution volumes (30 to 80 mL), i.e., a mass-to-volume ratio (m/v) increase from 1:30 to 1:10 to cause more supersaturation to the system and enhance the ASC-NIC cocrystal yield. For clarity, the masses of ASC and NIC used in GAS for BBD and optimization runs at each desired condition are shown in the Supporting Information (Table S1).

### 2.5 Cocrystal Characterizations

#### 2.5.1 Powder X-ray Diffraction (PXRD)

Crystalline identification of the material was obtained using a benchtop powder diffractometer (MiniFlex600, Rigaku, USA), equipped with a copper radiation source (*k*_alpha_: 1.54059 Å, 40 kV voltage and 15 mA current), divergence slit (1.25 °, 10 mm), and a D/teX Ultra detector. Measurements were taken at room temperature by angular scanning in the θ-2θ mode between 2 and 35° with a step size of 0.02° 2θ, a scanning speed of 10°/min and divergence slit (1.25 °, 10 mm). PXRD data was used without any preprocessing and diffractograms were created using Origin software (version 2021).

#### 2.5.2 Thermal Analysis (DSC/TGA)

Thermal analysis was performed using a simultaneous thermogravimetric-differential calorimeter scanner (STA 449 F3 Jupiter, Netzsch, Germany). Samples (4–10 mg) were placed in aluminum pans, inertized for 30 min prior analysis, and a heating ramp (20 °C-300 °C) was then applied under N_2_ (20 mL·min^–1^). Scans were run in a single heating cycle at a 10 °C·min^–1^ heating rate. Mass changes in the samples with temperature were recorded to detect the degradation path. The crystallinity index of cocrystals (*X*_*C*_) was determined based on the relation between fusion enthalpy for the samples (ΔH_f_) and the standard (ΔH_f_^0^) by integration of the cocrystal endothermic peak area (147.5 °C), according to eq 4.^43^

$$X_{C}=\frac{{\mathrm{\Delta H}}_{f}}{{\mathrm{\Delta H}}_{f}^{0}}$$ 4

The value for ΔH_f_^0^ for the ASC-NIC cocrystal was not available in the literature and was calculated as the mean value for the heat of fusion of cocrystals with 100% purity, which is 207.2 J·g^–1^.

#### 2.5.3 Scanning Electron Microscopy (SEM)

The morphology and particle size of the produced cocrystals were analyzed using a desktop scanning electron microscope (Hitachi TM3030, Japan) operated at 15 kV. The samples were adhered to double-sided carbon tape on the surface of stubs and then gold-coated before microscopy. SEM images with 2,500× magnification were converted to 8-bit files and employed for particle measurement. The mean characteristic length of particles (ca. 200) was measured with the open-source ImageJ software v. 1.53 (Bethesda, USA).^44^

#### 2.5.4 Antioxidant Activity (AA)

Three different AA assays were performed on the cocrystals to fully encompass possible different antioxidative mechanisms, namely (i) the DPPH method according to Brand-Williams, Cuvelier, and Berset,^45^ (ii) the ferric reducing antioxidant power (FRAP) method developed by Benzie and Strain,^46^ and (iii) the 2,2’-Azino-bis­(3-ethylbenzothiazoline-6-sulfonic acid (ABTS) radical assay.^47^ Trolox was the standard antioxidant used to obtain the calibration curves. All methods were adapted to a 96-well microplate scale; samples (10 mg) were diluted in distilled water to fit the calibration curve, and aliquots (50 μL) were added with 250 μL of each reactant separately. After 30 min of incubation in the dark, the absorbance was measured in triplicate in an Epoch microplate reader (BioTek, Agilent Technologies, USA). Results were expressed as Trolox equivalents (TE) per gram of ascorbic acid in the samples (mmol TE·g ASC^–1^), considering the actual content of ASC in each sample.

### 2.6 Quantification of Cocrystal Components and Stoichiometry

The final contents of ASC and NIC in cocrystal samples obtained by GAS were quantified through different techniques, namely first-order derivative spectrophotometry (FODS), high-performance liquid chromatography (HPLC), and elemental analysis to confirm the final stoichiometry regarding the ASC:NIC ratio in cocrystals, as well as the presence of impurities in the form of ASC or NIC homocrystals.

#### 2.6.1 First-Order Derivative Spectrophotometry (FODS)

The simultaneous determination of ASC and NIC in cocrystals was performed using the FODS method, as described in the literature (Balbinot Filho et al.;^16^ Biscaia et al., 2020 ^201^), on a T90+ UV–vis spectrophotometer (PG Instruments, UK). The samples were dissolved in sodium oxalate buffer (pH 5.3) to maintain ASC stability in aqueous media^48^ and then scanned in the ultraviolet region (200–400 nm) using 1 cm length quartz cuvettes. The absorbance at zero-crossing points for each pure compound in the first-order derivative spectra was recorded at 243 and 261 nm for NIC and ASC, respectively,^16^ and the results were obtained using two independent calibration curves (R^2^ > 0.997).

#### 2.6.2 High-Performance Liquid Chromatography (HPLC)

Analysis by HPLC-UV (Prominence LCMS 2020, Shimadzu, Japan) validated results found by the FODS method. The stationary phase consisted of a Luna 5 μm C18(2) 100Å column (Phenomenex) of 150 × 4.60 mm. An SDS[Fn fn1] mix (A) and acetonitrile (B) were used as the mobile phase in the isocratic mode (70%A/30% B) with injections (5 μL) at 0.3 mL·min^–1^ and 30 °C. Quantification of ASC and NIC was performed by measuring the peak area corresponding to detections at 244 and 262 nm (retention times of 4.80 and 5.56 min, respectively). Results expressed as relative (%) contents of ASC and NIC in the samples.

#### 2.6.3 Elemental Analysis

Elemental analysis was performed (PerkinElmer, Model 2400 Series II) to assist HPLC results and to define the molar proportion between ASC and NIC in cocrystal samples by calculating the relative percentage of carbon (C), hydrogen (H), and nitrogen (N) in the cocrystals.

### 2.7 Cytotoxicity Assay

#### 2.7.1 Cell Culture

Human umbilical vein endothelial cells (Huvec) were cultured in RPMI-1640 medium supplemented with 10% fetal bovine serum (Gibco, USA) and 1% penicillin/streptomycin (Thermo Fisher Scientific, USA). Cells were cultured in 96-well plates at a density of 50,000 cells/mL, with a final volume of 200 μL per well, and maintained in a humidified incubator at 37 °C with 5% CO_2_.

#### 2.7.2 MTT Assay

ASC-NIC cocrystals of high purity (>99%) obtained by GAS were employed in cytotoxicity assays, considering the theoretical molar mass of the 1:1 complex 298.24 g·mol^–1^ (NCBI, 2024 ^200^). Cells were incubated alone as the control sample and with the produced cocrystals (10–500 μmol·L^–1^) for a period of 24 h. After this period, the medium was removed from the wells, and 100 μL of an MTT solution in culture medium at a concentration of 5 × 10^–3^ g·mL^–1^ was added. After 3 h incubation, the MTT solution was removed from the wells, and the formed crystals were dissolved using 100 μL of DMSO in each well. Absorbance was then read in a spectrophotometer at a wavelength of 540 nm. Cell viability was calculated by comparing the samples treated with the cocrystals to the control, which yielded a cell viability of 100%.

### 2.8 Statistical Analysis

Experiments were performed in triplicate, and statistical differences between means at the 95% confidence level (α = 0.05) were determined using one-way Analysis of Variance (ANOVA). The Least Squares Differences (LSD) test was used to compare means across homogeneous groups.

## 3 Results and Discussion

### 3.1 Coformer Solubility in Ethanol

For proper cocrystallization by the GAS method, the selected solvent should dissolve the starting material in an appropriate amount.^37^ The equilibrium solubilities of ASC and NIC in ethanol were determined at atmospheric pressure, and the results are presented in Table 1. As expected, the solubility increased with temperature, and values (molar fractions) found are in agreement with studies on the solubility of ASC^35,49,50^ and NIC^34,51,52^ in organic solvents. At 25 °C, the NIC solubility was likely 1 order of magnitude higher than the ASC solubility, and this tendency continued at higher temperatures employed, following BBD. Similarly, the consulted literature reports that the NIC is approximately 13 times more soluble in pure ethanol at 25 °C compared to the ASC solubility.

**Table 1:** Solubility Mole Fraction of Vitamins C (l-Ascorbic Acid) and B_3_ (Nicotinamide) in Pure Ethanol at Increasing Temperatures

Temperature	l-ascorbic acid	Nicotinamide
25 °C	4.6 × 10^–3^ ± 0.001^a^	45.3 × 10^–3^ ± 0.021
35 °C	7.8 × 10^–3^ ± 0.001	71.9 × 10^–3^ ± 0.006
45 °C	10.8 × 10^–3^ ± 0.001	96.3 × 10^–3^ ± 0.007
55 °C	17.9 × 10^–3^ ± 0.004	107.5 × 10^–3^ ± 0.002

a Mean ± standard deviation. Calculated as $x_{1}=\frac{m_{1}/M_{1}}{m_{1}/M_{1}+m_{2}/M_{2}}$ . Where *m* represents the mass (g) and *M* is the molecular mass (g·mol^–1^) of ascorbic acid/nicotinamide (1) or ethanol (2).

Regardless of any cosolvency phenomena, the solubility of the solute in the solvent substantially decreases when in contact with CO_2_ due to the antisolvent effect (Balbinot Filho et al.^59^), which guarantees high supersaturation of the solutes and their coprecipitation as a cocrystal at higher rates. The solubility data for ASC and NIC in the CO_2_/ethanol mixture at high pressures are currently unavailable. Indeed, the coformer NIC is 100 times more soluble in neat supercritical CO_2_ (40 °C, 200 bar) than ASC,^53,54^ and therefore, the NIC solubility in a mixture of CO_2_/ethanol should not be negligible. Therefore, the solubility difference of the cocrystal parent compounds in the CO_2_/ethanol at GAS conditions reflects how compounds would distribute between the precipitate (cocrystal) and the fluid mixture vented out. As illustrated in Figure 1, ASC can precipitate as pure homocrystals, with the cocrystal and part of NIC partitions in the precipitate (as a cocrystal), but does not produce homocrystals due to its higher solubility. However, the yield and purity aspects of the cocrystallization of ASC and NIC with pressurized CO_2_ have not been reported.

**Figure 1:**
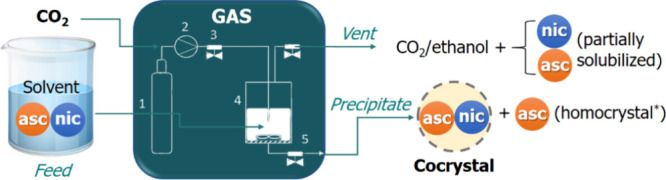
Illustration of cocrystallization of NIC (more soluble) and ASC (less soluble) noncongruent system in terms of solubility in ethanol by the GAS method. *Only for runs with a starting ASC content exceeding the cocrystal stoichiometry.

### 3.2 Box–Behnken Experimental Design (BBD)

#### 3.2.1 Cocrystal Identification

The results of PXRD diffractograms (Figure S1, Supporting Information) and DSC thermograms (Figure S2) analysis, conducted following BBD experiments, evidenced the formation of the 1:1 (ASC:NIC) cocrystal, as reported in previous publications by our research group,^16,17^ attempting to reproduce this cocrystal polymorph by GAS. PXRD was used to compare the structural fingerprints of the collected powders in BBD, the CSD-simulated pattern for the reference cocrystal (code OXOHEQ), and the pure compounds in the same plot. New intense reflection peaks at 2θ of 12.8°, 16.3°, 24.1°, and 28.55° (asterisks in Figure S1), absent in the ASC and NIC diffractograms, and observed at distinct positions for pure compounds (dashed lines), confirm the formation of a new crystalline structure. Notwithstanding, most DSC runs showed a unique endothermic event at an intermediate melting temperature of 147 °C concerning pure ASC and NIC, which is typical for the ASC-NIC 1:1 cocrystal. Therefore, the cocrystal purity and yield results from BBD assays are based on the 1:1 polymorph.

#### 3.2.2 Model Fitting and Variable Significance

The experimental design and the obtained responses for all 15 GAS runs and the optimal point (OP) are shown in Table 2, which presents the values for observed (experimental) versus predicted (model) responses. The adjusted quadratic models (R^2^ > 0.91) revealed that the independent variables explained more than 90% of the variation in the responses, with some minor unexplained variance.^55,56^ Table S2 (Supporting Information) presents the model’s coefficients, based on coded variables, and their significance within the evaluated confidence level. All models were significant (*p < 0.05*) at a 95% confidence level and did not have a significant lack of fit (*p > 0.05*) following ANOVA and *F-test* (Table S2), meaning that the selected models were acceptable in predicting the responses.^42,55^ Moreover, the models provided close predictions for responses obtained at the optimum GAS operational point (OP).

**Table 2:** Box–Behnken Experimental Design and Results for Predicted *versus* Observed Responses, as well as Optimized GAS Condition^a^

	Independent variables (factors)			Cocrystal purity (%)		Cocrystal mass yield (%)		*Mean length (*μ*m)*	
GAS run	T (°C)	*F (L·h* ^*‑1*^ *)*	M (ASC:NIC)	*Obs.*	*Pred.*	*Obs.*	*Pred.*	*Obs.*	*Pred.*
#1	35	0.6	1:1	92.95 ± 1.69^b^	93.28	68.65 ± 1.06^b^	66.91	11.32 ± 0.44	11.32
#2	55	0.6	1:1	87.97 ± 0.35^c^	86.53	36.23 ± 0.16^g^	33.71	4.05 ± 0.15	4.05
#3	35	0.9	1:1	98.91 ± 0.02^a^	100.34	46.48 ± 0.07^e^	49.00	7.49 ± 0.44	7.49
#4	55	0.9	1:1	93.32 ± 1.81^b^	92.98	47.52 ± 0.98^e^	49.26	10.92 ± 0.47	10.92
#5	35	0.6	1:2	99.61 ± 0.27^a^	106.05	67.01 ± 0.01^b,c^	70.25	9.40 ± 0.6	9.40
#6	55	0.6	1:2	71.47 ± 2.97^e^	79.69	62.86 ± 1.21^g^	66.89	12,75 ± 0.55	12.75
#7	35	0.6	2:1	55.08 ± 2.15^g^	46.87	66.91 ± 2.69^b,c^	62.88	7.30 ± 0.4	7.30
#8	55	0.6	2:1	65.57 ± 2.62^f^	59.12	36.53 ± 1.06^f^	33.29	7.38 ± 0.43	7.38
#9	45	0.3	1:2	100.25 ± 0.17^a^	93.47	63.70 ± 0.03^b,c,d^	62.19	9.34 ± 0.52	9.34
#10	45	0.9	1:2	99.59 ± 0.3^a^	91.71	66.96 ± 0.01^a^	61.20	13.03 ± 0.58	13.03
#11	45	0.3	2:1	37.19 ± 1.9^h^	45.07	36.13 ± 1.83^f^	41.89	8.67 ± 0.47	8.67
#12	45	0.9	2:1	53.58 ± 2.35^g^	60.36	39.02 ± 1.69^f^	40.53	8.67 ± 0.47	9.13
#13	45	0.6	1:1	97.38 ± 0.28^a^	92.70	58.37 ± 2.48^d^	60.19	6.27 ± 0.36	6.01
#14	45	0.6	1:1	86.43 ± 0.84^d^	92.70	61.41 ± 5.23^c,^ ^d^	60.19	5.29 ± 0.32	6.01
#15	45	0.6	1:1	94.29 ± 0.01^a^	92.70	60.80 ± 2.58^c,d^	60.19	6.48 ± 0.34	6.01
OP^2^	37.8	0.58	1:1.12	99.85 ± 1.03^a^	100.0	65.71 ± 4.23^b,c^	66.69	7.30 ± 0.61	6.81

a ^1^Variables: Temperature (*T*), CO_2_ flow rate (*F*), and ASC to NIC molar ratio (*M*). ^2^Optimum operational point. ^a,b^Means followed by different letters in the same column are significantly different (*p* < 0.05) by the LSD test.

Significant and nonsignificant effects of temperature (*T*), CO_2_ flow rate (*F*), and molar ratio (*M*) of compounds on the dependent variables are detailed in Table 3. It is noteworthy to mention that the coformer (ASC to NIC) molar ratio (*M*) had a significant (*p* < 0.05) decreasing effect on all evaluated responses, as indicated by the negative values of the regression coefficients (Table S2). These influences on the responses are briefly discussed below.

**Table 3:** Significance (*p-*Value) of Linear, Quadratic, and Cross Factors on BBD^a^

Effect	Purity	Yield	Length
*Model*	0.0033**	0.0171*	0.0322*
*Temperature (T)*	NS	0.0047**	NS
*Temperature (T* ^2^ *)*	NS	NS	NS
*Flow rate (F)*	NS	NS	NS
*Flow rate (F* ^*2*^ *)*	NS	0.0092**	0.0386*
*Molar ratio (M)*	0.0025**	0.0031**	0.0229*
*Molar ratio (M* ^*2*^ *)*	0.0205*	NS	0.0185*
*TxF*	NS	0.0091**	0.0138*
*TxM*	NS	0.0147*	NS
*FxM*	NS	NS	NS

a *Significant factors at the 95% confidence level (*p* < 0.05), and **99.5% level (*p* < 0.005).

#### 3.2.3 Cocrystal Purity and Yield

The cocrystal phase purity and yield are crucial factors to consider when designing a cocrystallization process, especially for systems with noncongruent solubility.^23,27^ The coformer ratio (*M*) was the only factor significantly affecting cocrystal purity (37% to 100%), while linear and interaction effects of temperature also had a marked impact on cocrystal yield (36–68%). Other studies have also reported the importance of the ratio of the starting components affecting the final properties of cocrystals produced by the GAS method.^39,42^

Previous reports for GAS cocrystallization^16,17,30,37,40^ showed that a cocrystal’s definite stoichiometry is maintained despite an excess of the more soluble cocrystal component in the starting solution. This fact is corroborated by noticing PXRD patterns (Figure S1, Supporting Information) for GAS runs held at an initial *M* of 1:1 (#1, #2, #13, and #15) are the same for those starting from an *M* of 1:2 (#5 and #10). Therefore, no trace of residual NIC was detected in the cocrystals since the excess NIC was under the solubility limit to precipitate as homocrystals,^30,40^ and powders were cocrystal-pure.

At the same time, when the ASC content exceeds the stoichiometric 1:1 cocrystal in the feed solution, part of the excess vitamin C remains in the produced cocrystal, reducing its purity. This behavior is evident through peaks at 2θ values of 19.9 °, 28.1 °, and 30.1 ° for runs #7, #8, #11V and #12 in PXRD (dashed lines in Figure S1), corresponding to pure ASC. According to DSC (Figure S2), purer GAS-cocrystals (purity >90%) showed a unique endothermic peak at 146–147 °C, characteristic of the ASC-NIC cocrystal.^16,57^ In contrast, less pure cocrystals presented ASC homocrystals, detected as minor peaks between 193 and 196 °C, or with elongated melting peaks (red details in Figure S2b).^58^ Therefore, the combined PXRD and DSC results corroborate the lower purity of the cocrystal batches from runs #7, #8, #11, and #12, which were produced from a solution with a 2:1 (ASC to NIC) molar ratio.

The 1:1 ASC-NIC cocrystal (298.25 g·mol^–1^) has a 59.1 wt % content of ASC and 40.9%wt of NIC, which is closely related to the composition and final stoichiometry shown in Table 4 for the purest cocrystals as determined by HPLC and FODS method. The data set shows a close agreement between the two analytic techniques (HPLC and FODS), and atomic composition by elemental analysis confirms that a 1:1 stoichiometric cocrystal of high purity (>99%) was obtained for runs starting from a 1:1 or 1:2 (ASC:NIC) ratio. Pure cocrystal samples also showed a high crystallinity index (>0.9), determined from DSC endotherms (Table 4), indicating a high degree of organization in the crystalline packing for the ASC-NIC cocrystal with minor amorphous regions.

**Table 4:** Composition of Precipitated Powders by GAS-BBD, Stoichiometric Ratio, Elemental Analysis, and Crystallinity Index

	HPLC (% wt)		FODS (% wt)			**Elemental analysis**(% wt)^a^			
GAS run	ASC	NIC	ASC	NIC	Molar ratio	*H*	*C*	*N*	X_c_ ^b^
#1	59.1	40.9	60.4	39.6	1:1.0	47.79	4.75	9.06	1.00
#5	58.7	41.3	59.1	40.9	1:1.0	48.25	4.81	9.43	1.01
#7	75.4	24.6	76.2	23.8	2.1:1	44.66	4.71	5.06	
#9	57.7	42.3	58.9	41.1	1:1.1	48.26	4.75	9.53	0.99
#11	78.3	21.7	83.6	16.4	3.6:1	42.91	4.57	2.96	
#13	58.7	41.3	59.1	40.9	1:1.1	47.54	4.80	8.26	0.90
#15	58.9	41.1	59.7	40.3	1.1:1	47.62	4.69	8.96	1.01

a Reference values for the 1:1 cocrystal (C_12_H_14_N_2_O_7_) ^8,14^: C (48.32%), H (4.73%), and N (9.39%).

b Crystallinity index (DSC).

Despite the loss during the collection and transfer of the micronized powders (MacEachern et al.^27^), the residual solubility of the components impacts the GAS yield. The temperature effect on cocrystal yield is directly related to the increase in solubility in CO_2_/ethanol at constant pressure.^59^ GAS runs performed at 55 °C (#2, #4, and #8) had overall low yields, below 50% wt. Moreover, the process at higher temperatures contributes to the ASC degradation. Analogously, Dias et al.,^37^ studying quercetin-nicotinamide cocrystals, observed lower cocrystal yields in GAS method due to the loss of NIC through solubilization in CO_2_/acetone. Nonetheless, significant (*p* < 0.05) cross-interaction effects of temperature (*TxF* and *TxM*) on cocrystal yield are represented by RSM (Figure S3), revealing complex relationships between the studied factors , which help to understand the lower yields also obtained at 35 °C (#3) and at 45 °C (#11 and #12).

#### 3.2.4 Cocrystal Morphology and Particle Size

The mean length of GAS-cocrystals (4–13 μm) was mainly affected by a *TxF* synergistic (positive) interaction, leading to longer particles as both factors increased, as seen in the RSM of Figure S4. As supersaturation is the driving force for crystallization, increasing the process temperature can affect the particle size distribution (PSD) due to the agglomeration of large particles resulting from the increased solubility.^20^ The negative quadratic effect of the process CO_2_ flow rate (*F* ^2^) was also significant (*p* < 0.05), indicating a nonlinear dependence of cocrystal lengthtwith*F*, characterized by the presence of regions of maxima and minima. The increase in the antisolvent flow rate also causes turbulence to the system, resulting in enhanced mixing and rapid supersaturation and nucleation, which generate finer particles compared to a low CO_2_ flow rate.^20,27,40^ Therefore, adjustments in process variables, such as temperature, coformer concentration in the solution, and antisolvent flow regime, can be used to tune the particle size of the produced materials.^27^

The morphology of BBD cocrystals can be visualized in SEM micrographs at 2,500× magnification (Figure 2). Some GAS runs produced thin, elongated plates (#1, #10, #15), which are regarded as needles of uniform width and varying length, and can be attributed to a purer cocrystalline phase, unlike the larger blocks obtained in less pure samples (#8, #11, and #12). Some samples presented mixed morphologies (#3, #9, #14) and some degree of particle agglomeration (#2, #7). Padrela et al.^28^ reported that needle-shaped crystals result from the antisolvent mechanism, while blocks are formed when solvent extraction from liquid droplets into the supercritical media is favored. Therefore, these combined morphologies can be due to concurrent crystallization mechanisms at the varying conditions employed in BBD experiments.

**Figure 2:**
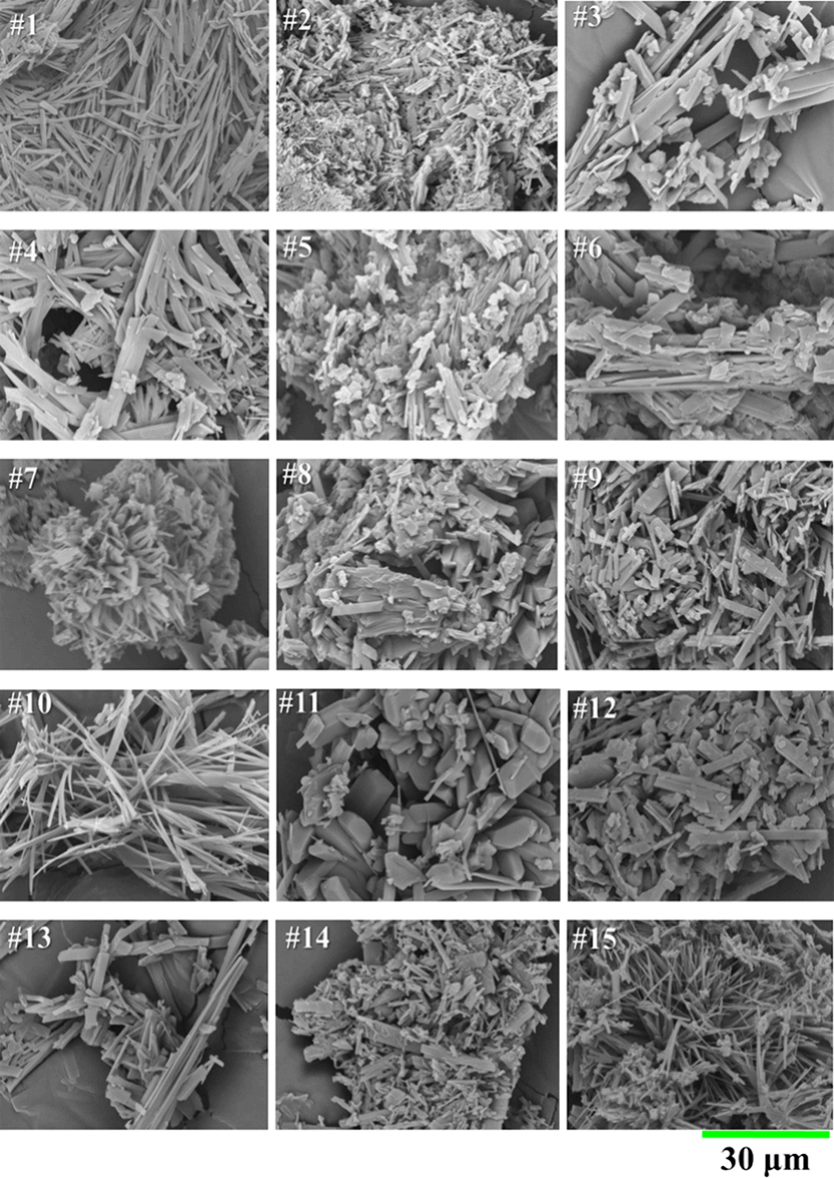
SEM micrographs (2,500x resolution) of GAS-cocrystals obtained in BBD. Note: Numbers following hashes (#) refer to samples obtained from GAS-BBD experiments. For reference, see Table 2.

#### 3.2.5 Desirability Profiler and Optimal Point (OP)

Multiresponse designs increase the complexity of the models generated for optimizing multiple responses. Therefore, the desirability tool can contribute to establishing the optimal conditions for the best process performance, based on user-defined specifications of factors, and observing the response variation of BBD. The built desirability profiler (Figure 3) simultaneously optimized the responses regarding the cocrystal’s purity and yield (maximize) and particle length (minimize), and the returned optimal point (OP), considering all responses was 37.8 °C, 0.58 L·h^–1^ CO_2_ flow rate, and a 1:1.12 molar ratio (ASC:NIC). Therefore, the optimized value for molar ratio (*M,* 1:1.12) means that cocrystal precipitation occurs from a nonstoichiometric solution of input constituents since nonequivalent concentrations of coformers are used to attenuate the noncongruent solubility.^23,27^

**Figure 3:**
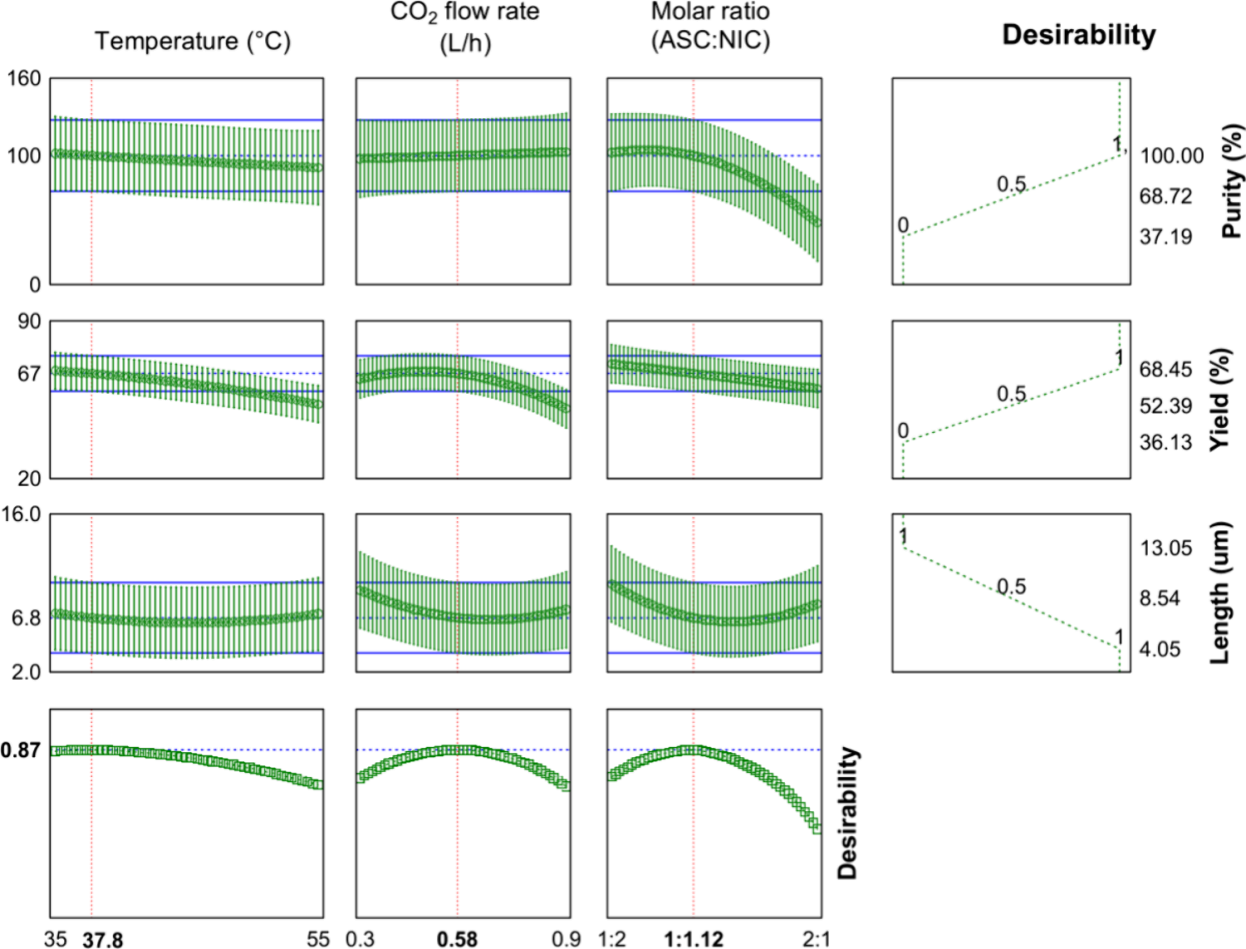
Desirability profiler and optimum point for the ASC-NIC cocrystallization by GAS.

The desirability value at OP was high (0.87 out of 1.0), and 2D (contour) and 3D (surface) plots of desirability as a function of cross-parameter interactions are shown in Figure S5, where regions of high desirability (>0.8, dark red) with the variations of *T*, *F*, and *M* can be observed. Predicted *versus* observed mean responses for GAS runs performed at OP are compared in Table 2. The good agreement between each response at optimized conditions validates the models and demonstrates that GAS can be a reproducible method for obtaining cocrystals with modulated characteristics. However, the production optimization approach to these methods is overlooked.^27^ For example, GAS cocrystals at optimized conditions through BBD had faster dissolution rates than those produced by conventional methods.^42^

### 3.3 Optimization of Cocrystallization Yield by GAS

The recovery percentage in cocrystallization methods using supercritical fluids is often less than 50%. Considering the factors (*T*, *F*, and *M*) within the limits employed in BBD, the maximum observed cocrystal yield was 67% of 70%, as predicted by the model (Table 2). However, unlike conventional crystallization, pressure is a factor that affects both solubility and supersaturation in pressurized systems.^27^ Since pressure was not a variable from the start, it was slightly reduced from 90 to 80 bar in GAS experiments to maintain a condition above the critical point of CO_2_ (31 °C and 73.8 bar) and close to the pressure of minimal relative volume expansion of the CO_2_/ethanol mixture that occurs at 77 bar and 40 °C.^60^ Various studies have shown that using operational pressures close to this point is favorable for the complete recuperation of precipitates by GAS, even within a very narrow pressure window.^61–63^

In addition, the molar concentration of the solutes (mol/L) in the feed solution was progressively increased from 1:30 to 1:10 (see Table S1), observing the 1:1.12 (ASC:NIC) molar ratio to accelerate the supersaturation during GAS while keeping all other parameters (*T, F, M*) fixed at OP. A progressive yield increase up to 85.24% was obtained at 80 bar and 1:10 (m/v), as shown in Figure 4. As a result, cocrystal recuperation increased 10-fold (from 0.2 to 2 g), with cocrystal purity maintained at nearly 100%. Cuadra et al.^31^ observed a 15% yield increase in the precipitated cocrystal when the pressure was reduced by 50 bar, and a higher yield was observed with increased coformer concentration in the solution.^40^ Therefore, the material loss in the GAS process can be mitigated by adjusting scalable variables that enhance supersaturation, such as lowering pressure and increasing coformers concentration in ethanol.

**Figure 4:**
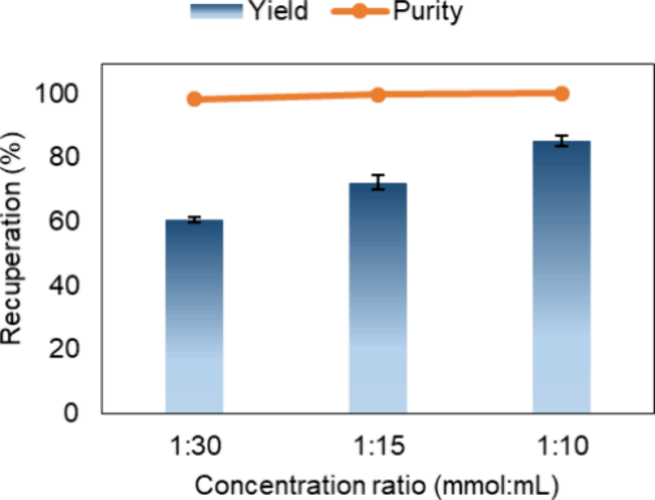
Cocrystal yield and purity for GAS runs performed at 80 bar and different mass-to-volume ratios.

### 3.4 Thermal Stability

DSC/TGA thermograms for the cocrystal obtained at OP (Figure 5) show negligible mass loss before the melting point, confirming that the structure was preserved until the onset of decomposition (approximately 160 °C), as evidenced by the absence of new peaks and the retention of existing ones.^64^ Moreover, there was no significant degradation of vitamin C in the cocrystal exposed to heat (80 °C) for 24 h, as measured by the ASC content in the exposed samples at regular time intervals (data not shown). The good chemical stability of ASC as a cocrystal with NIC in powder form is ascribed to the hydrogen bonding and protection of reactive hydrogens in the enediol group of ASC that causes its self-oxidation.^7,65^ ASC cocrystals also remained stable during prolonged storage, exhibiting improved photostability.^3,66^ The organized arrangement and periodicity in the crystal lattice causes longer distances between reactive sites, thus eliminating the photodegradation mechanism.^67^

**Figure 5:**
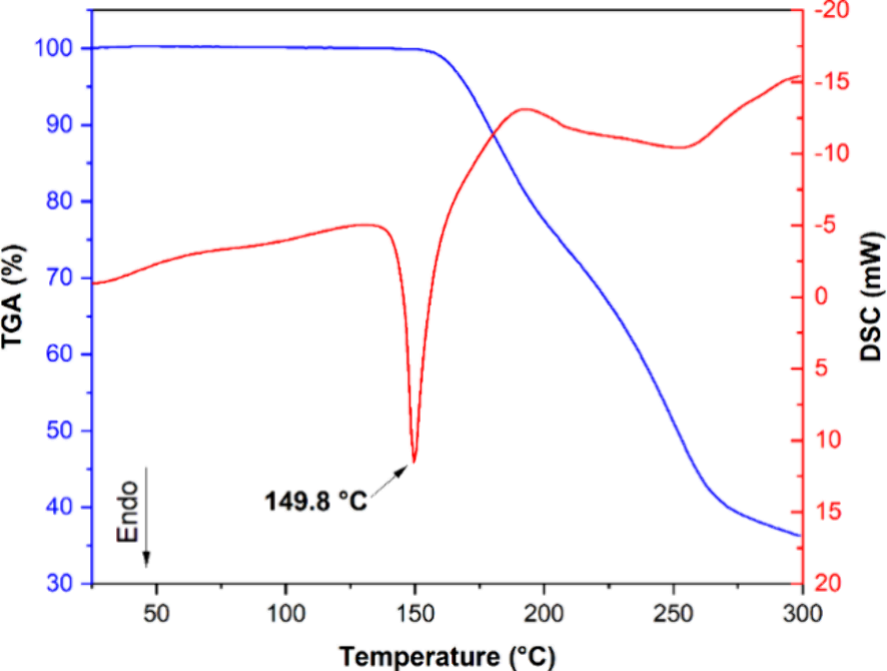
DSC/TGA thermograms for GAS-OP cocrystal.

### 3.5 *In Vitro*Potential of ASC-NIC Cocrystals

#### 3.5.1 Antioxidant Activity (AA)

The antioxidant power of ASC as a cocrystal with NIC was evaluated *in vitro* for GAS-BBD cocrystals of higher purity (Figure 6). The AA of cocrystals was normalized on vitamin C content (59.1 wt %) to compare with pure ASC. The results showed an overall equal or slightly lower AA for the cocrystals compared to the pure vitamin, as detected by the ABTS method (3.7–6.2 mmol TE·g ASC^–1^, Figure 6a) and by the DPPH assay (4.1–7.9 mmol TE·g ASC^–1^, Figure 6b). For the FRAP method (Figure 6c), the AA of the cocrystals (3.5–4.8 mmol TE·g ASC^–1^) was mostly higher than that of pure ASC (3.8 mmol TE·g ASC^–1^). Such differences can be due to a pro-oxidant mechanism of ASC induced by Fe^2+^ ions in FRAP^68^ and a distinct antioxidant mechanism between the evaluated assays (electron transfer and hydrogen atom donation). Therefore, one can affirm that the produced cocrystals preserve most of the antioxidant potential of ASC since pure NIC did not present quantifiable AA by the tested methods. There was no synergism with NIC contributing to the AA of the ASC-NIC cocrystals, as also observed by Stolar et al.^9^ for the same cocrystal and its pure components.

**Figure 6:**
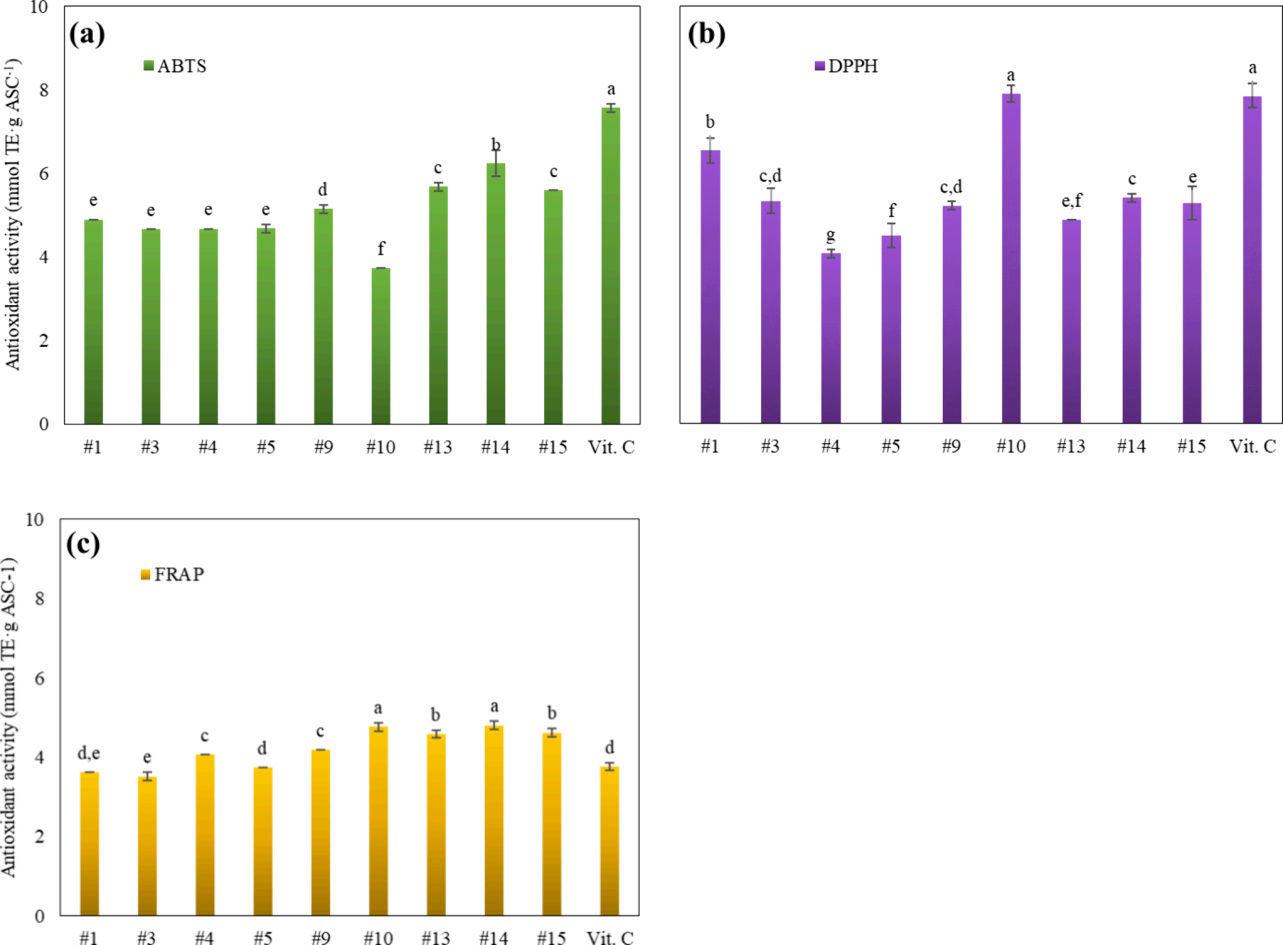
Comparison of the antioxidant activity of ASC-NIC GAS cocrystals and pure vitamin C by (a) DPPH, (b) ABTS and (c) FRAP assays. ^a,^ ^b^Means with different letters within the same method are statistically (*p* < 0.05) different by the LSD test.

#### 3.5.2 Cytotoxicity Assay

The cytotoxic potential of GAS cocrystals produced at OP was compared to that of the conventional LAG (liquid-assisted grinding) method, as determined after 24, 48, 72, and 96 h of incubation using the MTT assay. None of the tested cocrystals exhibited cytotoxicity upon exposure to healthy Huvec cells up to 96 h compared to the control. Only at higher concentrations (500 μM), above the range normally tested for cocrystals (3–30 μM),^69^ there was a slight reduction in cell viability observed after 96 h of incubation, of approximately 15%. (Figure 7). No significant reduction in cell viability was observed for the shorter incubation times, even at a concentration of 500 μM, as shown in Figure S6 of the Supporting Information. These results provide strong evidence for the safety of ASC-NIC cocrystals in healthy human cells, even when used at higher concentrations that are typically cytotoxic. Since cell viability percentages were above 80%,^69^ this preliminary result is unprecedented. It emphasizes that ASC-NIC cocrystals are not harmful to human skin cells, even at high concentrations, suggesting their safe use in topical applications.

**Figure 7:**
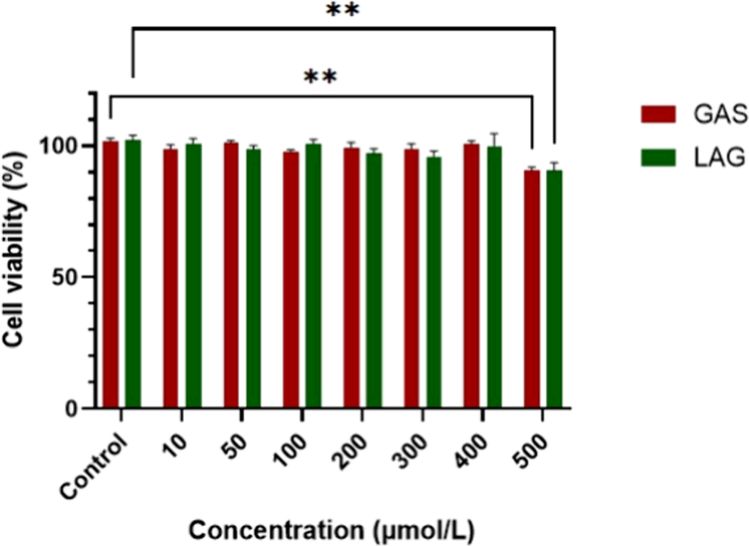
Cytotoxicity assay (after 96h incubation) for the cocrystal obtained by GAS at the optimum point and comparison to a cocrystal obtained by conventional liquid-assisted grinding (LAG) with ethanol. *Denotes significant differences (*p* < 0.005) between untreated (control) and treated samples. GAS and LAG refer to cocrystals obtained by gas antisolvent and liquid-assisted grinding methods, respectively.

Vitamin C is more cytotoxic to tumoral cells than it is to healthy cells, and numerous studies demonstrated positive results upon intravenous administration of high doses of vitamin C (3 to 10 mM) on inhibition of diverse tumoral cells.^70–72^ Vitamin C causes an imbalance in the redox state in cancer cells, inducing apoptosis by reducing the levels of intercellular ROS that promote DNA mutation.^72^ In another study, ASC attenuated the cytotoxicity of betulinic acid by 15% as a cocrystal, resulting in inhibition of HaCat cells.^73^ Our recent work^17^ demonstrated that cocrystals produced by GAS exhibited a more intense antioxidative mechanism at acidic pH (1.0) compared to neutral pH, which is attributed to the good stability of vitamin C in an acidified medium. Therefore, the vitamin C cocrystal with nicotinamide has potential applications for *in vivo* investigations, as it is safe for healthy cells and toxic to tumoral ones, and its mechanism can be accentuated at lower pH levels, suggesting a potential auxiliary therapeutic agent for targeted applications, such as gastric cancer.

## 4 Conclusions

The metabolically active nutrients vitamin C (l-ascorbic acid, ASC) and vitamin B_3_ amide (nicotinamide, NIC) can form cocrystals. This underexplored and potentially beneficial molecular adduct combines the advantageous properties of both substances within the same crystal lattice. Cocrystallization via high-pressure methods, such as the gas antisolvent (GAS) technique, makes an innovative route for the cocrystallization of these vitamins. This study focused on the production optimization of ASC-NIC cocrystals (1:1) by GAS with CO_2_ as an antisolvent and ethanol as a GRAS solvent. However, it was shown that ASC and NIC present distinct solubilities in pure ethanol, which, *a priori*, can hinder the cocrystal yield by GAS.

GAS produced high cocrystal-pure powders with fine particle sizes. The Box–Behnken design results showed that the ASC to NIC molar ratio in the feed ethanolic solution was the most significant factor affecting all responses. At optimized conditions, the cocrystal yield can be modulated to compensate for the solubility incongruence of the cocrystal parent compounds in the solvent by adjusting the process pressure and the mass-to-volume ratio of the compounds in the solution. The optimization strategy mitigated material losses and increased the maximum cocrystal yield by 23%, thereby improving GAS feasibility and surpassing yields typically achieved in cocrystallization by high-pressure methods, while maintaining the high purity of the cocrystals (>99%). Therefore, the limitation of solubility discrepancies of cocrystal components encountered in conventional low-pressure solution-based cocrystallization methods can be overcome in GAS, thereby expediting the process and conveying maximum purity and higher yields.

Moreover, the obtained cocrystals were thermally stable in the dry powder form, retained the antioxidant activity of vitamin C, and presented no *in vitro* cytotoxicity to healthy human epithelial cells, even at higher concentrations. GAS proved to be reproducible, scalable, and effective in producing ASC-NIC cocrystals that can be applied in the pharmaceutical field, where high-purity ingredients are required, or in the food industry as an additive or functional vitamin supplement. Based on the innovative aspects of the GAS cocrystallization at optimal operational conditions defined in this work, future studies should investigate the *in vivo* stability of these vitamin cocrystals against the various barriers of the human body. Moreover, pure vitamin C is selectively cytotoxic to tumor cells, and possible synergistic interactions with NIC should be explored to widen the opportunities for ASC-NIC cocrystals as a potential cancer treatment.

## Supplementary Material



## Footnotes

1 Mix A: Formate buffer (pH 3.5, 25 mM)/sodium dodecyl sulfate 5 mmol/L in acetonitrile (8:2 v/v).

## Notes

Complementary statistic data on the optimization experiments and ANOVA, PXRD diffractograms and DSC thermograms for Box–Behnken experiments, and 3D surfaces for significant parameter cross-interactions (PDF)
