# Supplementary material for: An Optimization Approach for the Production of High-Purity Vitamin C‑Nicotinamide Cocrystals by the Gas Antisolvent (GAS) Technique with CO2 and Ethanol
Source: ACS Omega. 2026 Jan 21;11(4):5352–63. doi: 10.1021/acsomega.5c08253 (PMC12878501; doi:10.1021/acsomega.5c08253)

Supporting Information

An optimization approach for the production of high-purity vitamin C-nicotinamide cocrystals by the gas antisolvent (GAS) technique with CO_2 and ethanol

Clóvis A. Balbinot Filho[a,*], Thayli R. Araujo[a], Jônatas L. Dias[a], Evertan A. Rebelatto[a], Adailton J. Bortoluzzi[b], Mariana M. Vernaschi[c], Tânia B. Creczynski-Pasa[c], Sandra R. S. Ferreira[a], Marcelo Lanza[a]

[a]*Department of Chemical Enginerring and Food Engineering, Federal University of Santa Catarina,*

[b]*Department of Chemistry, Federal University of Santa Catarina*

[c]*Department of Pharmaceutical Sciences, Federal University of Santa Catarina*

UFSC, 88040-900, Florianópolis, SC, Brazil

clovis.filho@posgrad.ufsc.br *corresponding author

Table S1. Masses of L-ascorbic acid (ASC) and nicotinamide (NIC) used in GAS at different ASC:NIC molar ratios (BBD) and mmol-to-volume ratios.

	**BBD[1] experiments**			**Optimization**		
Molar ratio (ASC:NIC)[2]	1:2	1:1	2:1	1:1.12		
ASC mass (g)	0.176	0.176	0.352	0.352	0.705	1.409
NIC mass (g)	0.244	0.122	0.122	0.259	0.547	1.094
Scale (mmol)	1	1	1	2	4	8
Solution volume (mL)	30	30	30	40	60	80
Concentration ratio (mmol:mL)	1:30	1:30	1:30	1:20	1:15	1:10

[1]Box-Behnken experimental design. [2]Values are based on the 1 mmol scale for a molecular mass of 176.12 g·mol^{-1} (ASC) and 122.12 g·mol^{-1} (NIC).

Table S2. Model's coefficients, significance (α = 95%), and fitting parameters by ANOVA.

**Factors[1]**	**Model coefficients for responses (effects)[2]**		
	**Cocrystal purity**	**Cocrystal yield**	**Particle length**
Main	0.9270*	0.6019*	6.0133*
(1) T(L)	-0.0353	-0.0824*	-0.0513
T(Q)	0.0043	-0.0180	0.7983
(2) F(L)	0.0338	-0.0059	0.8988
F(Q)	0.0015	-0.0867*	1.6333*
(3) M(L)	-0.1994*	-0.1024*	-1.5050*
M(Q)	-0.2020*	-0.0007	2.3958*
(1)x(2)	-0.0015	0.0837*	2.6750*
(1)x(3)	0.0966	-0.0656*	-0.8175
(2)x(3)	0.0426	-0.0009	-0.8075
**Model fit parameters (ANOVA)**			
R^2	0.9141	0.9346	0.9112
$F_{model}>F_{tab}$[3]	16.43	7.96	5.92
p_{model}	0.0033	0.0171	0.0322
$F_{lack\ of\ fit}<F_{tab}$[4]	4.58	18.45	6.45
$p_{lack\ of\ fit}$	0.1843	0.0518	0.1371

*Significant factors ($p < 0.05$). [1]Factors: Temperature (*T*), CO_2 flow rate (*F*), and ASC to NIC molar ratio (*M*). [2]Models built for coded effects (factors). [3]$F_{critical\ (9,5)} = 4.77$. [4]$F_{critical\ (3,2)} = 19.16$.

Figure S1. PXRD patterns for ASC-NIC cocrystals obtained in GAS-BBD experiments and comparison with pure compounds and cocrystal standard (code OXOHEQ)[1].

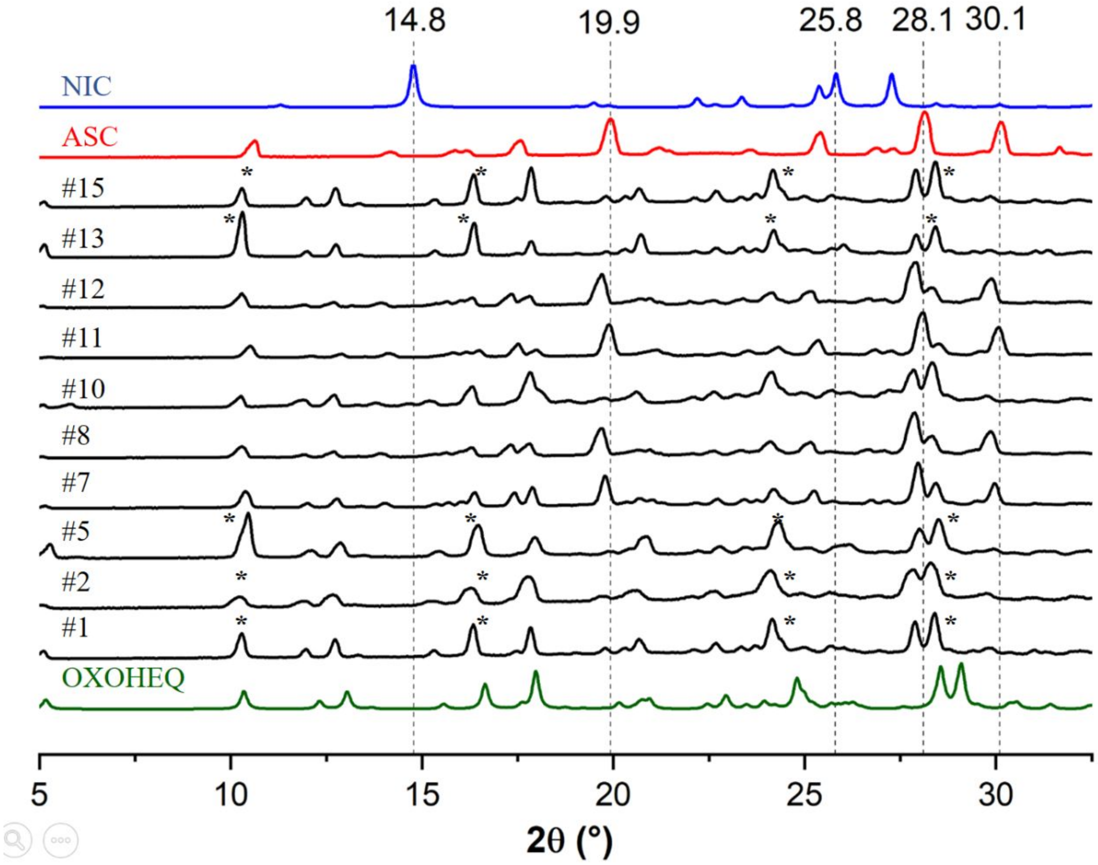

[1]Note: Vertical dashed lines represent typical diffraction angles of pure compounds (ASC and NIC), and asterisks mark new diffraction peaks in cocrystal samples.

Figure S2. DSC thermograms for GAS-BBD runs performed at ASC to NIC molar ratios of **(a)** 1:1 and 1:2 and **(b)** 2:1 and 1:1 (central point).

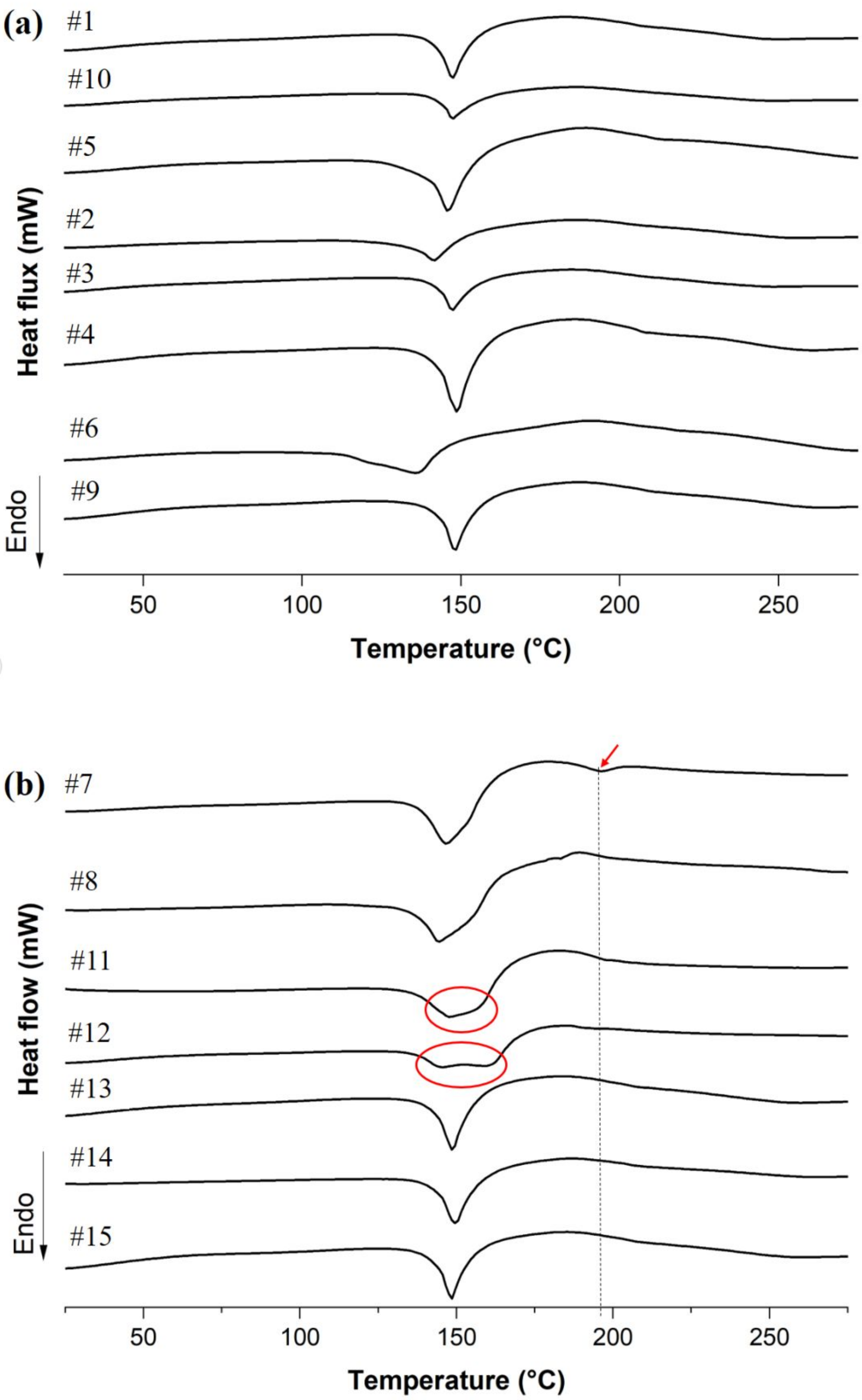

Figure S3. RSM for significant temperature interactions with CO_2 flow rate (M = 1.0) and ASC to NIC molar ratio (F = 0.6 mL·min^{-1}) for response cocrystal yield.

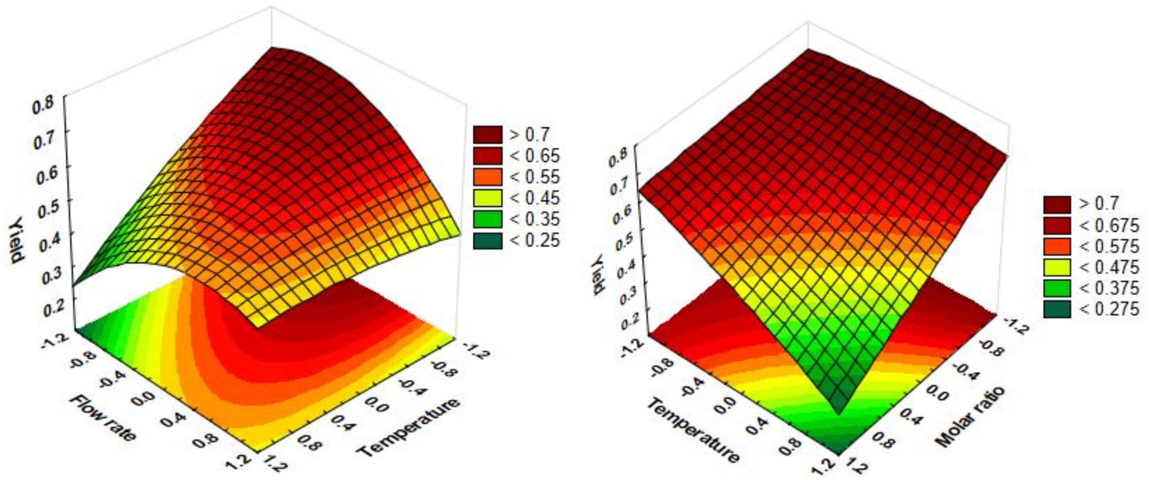

Figure S4. RSM for temperature versus CO_2 flow rate cross-interactions at fixed molar ratio (M = 1.0) for response cocrystal particle length.

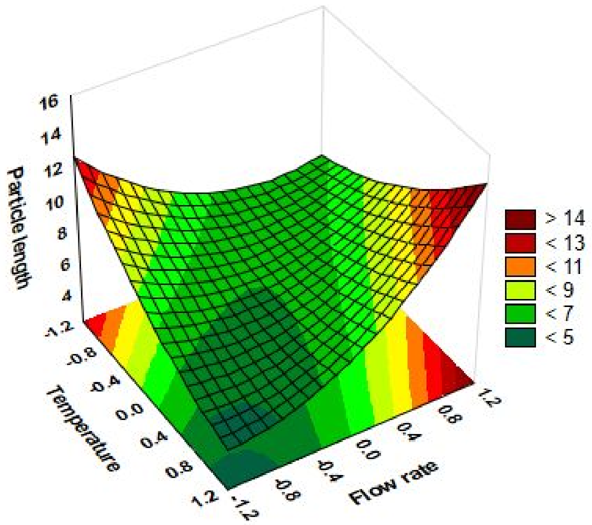

Figure S5. Desirability function surface plots for cross-parameter interactions *TxF*, *TxM* and *FxM*.

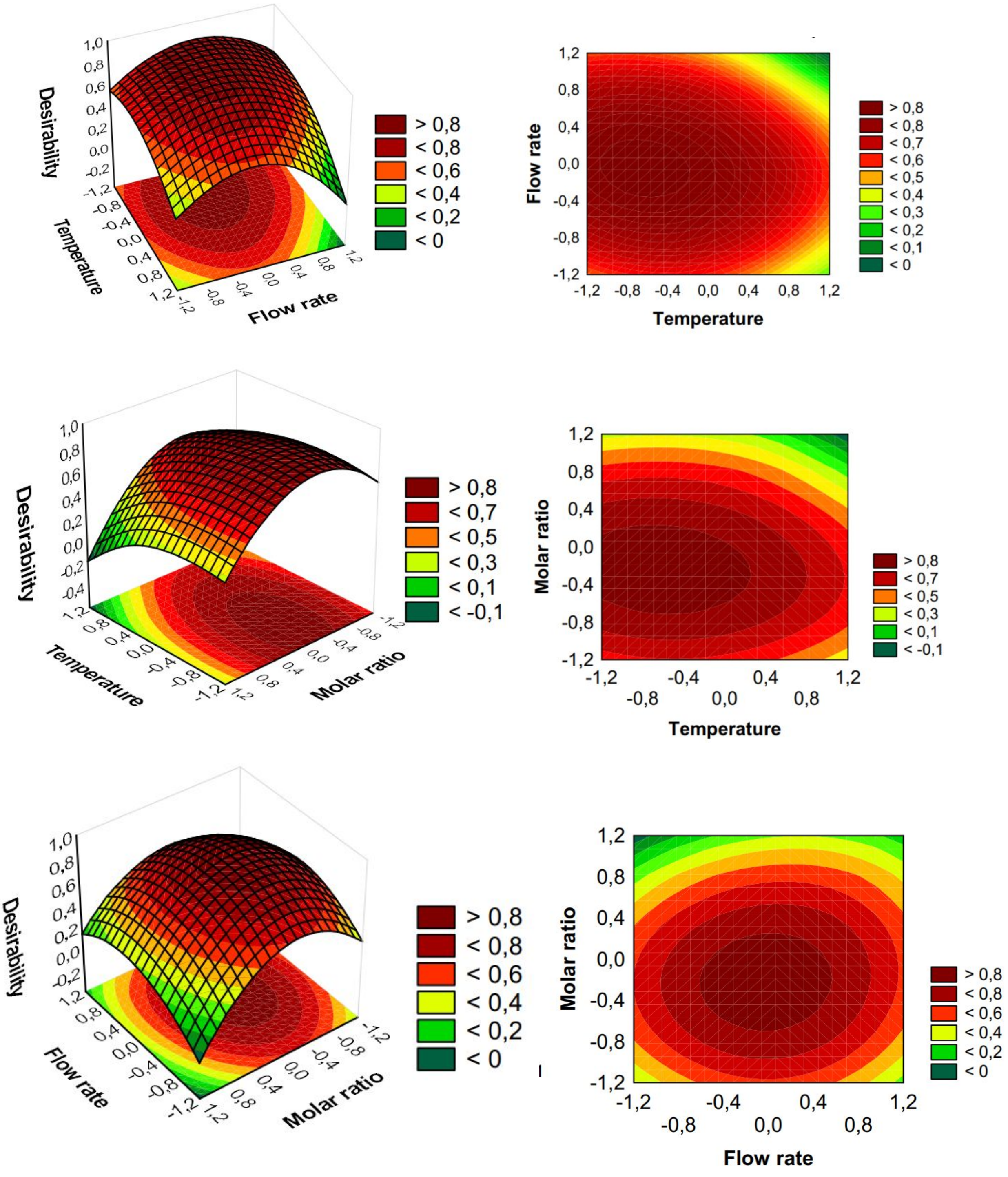

Figure S6. Cytotoxicity assay for the ASC-NIC cocrystal obtained by GAS (OP) and LAG at incubation times of **(a)** 24 h, **(b)** 48 h, and **(c)** 72 h.

(a)

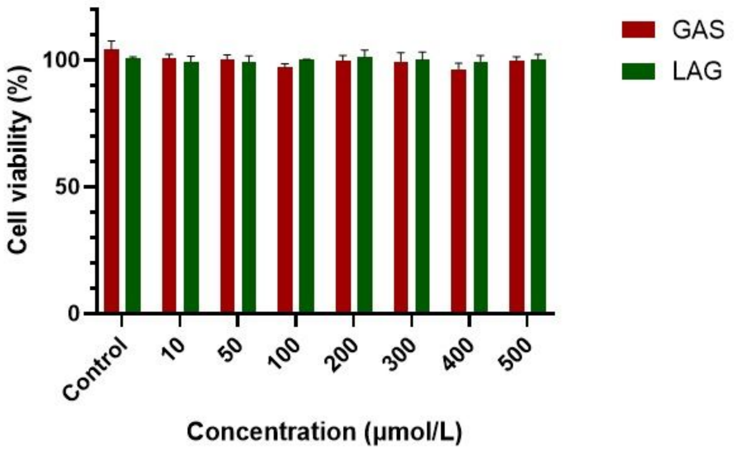

(b)

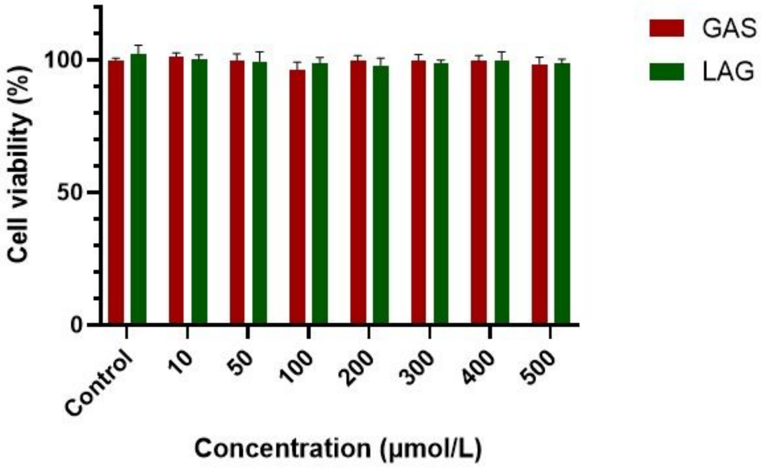

(c)

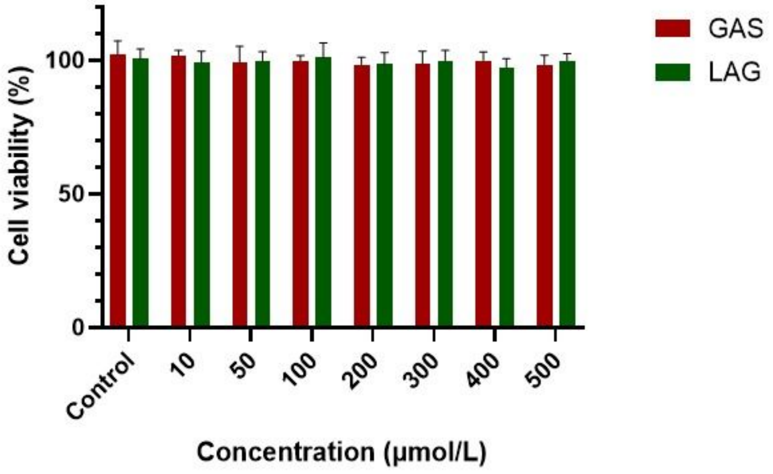

Supplement: Supplementary file 1 [file ao5c08253_si_001.pdf]
